# BCKDK Promotes Ovarian Cancer Proliferation and Migration by Activating the MEK/ERK Signaling Pathway

**DOI:** 10.1155/2022/3691635

**Published:** 2022-04-22

**Authors:** Huashun Li, Dongyang Yu, Lianbing Li, Juanjuan Xiao, Yijian Zhu, Yi Liu, Li Mou, Yafei Tian, Linbo Chen, Feng Zhu, Qiuhong Duan, Peipei Xue

**Affiliations:** ^1^Department of Pathology, The First People's Hospital of Tianmen, Tianmen, Hubei 431700, China; ^2^Department of Clinic Laboratory, Wuhan Children's Hospital (Wuhan Maternal and Child Healthcare Hospital), Tongji Medical College, Huazhong University of Science and Technology, Wuhan, Hubei 431700, China; ^3^NHC Key Laboratory of Birth Defects and Reproductive Health, Chongqing Population and Family Planning Science and Technology Research Institute, Chongqing 400000, China; ^4^Cancer Research Institute, The Affiliated Hospital of Guilin Medical University, Guilin, Guangxi 541001, China; ^5^Department of Biochemistry and Molecular Biology, School of Basic Medicine, Huazhong University of Science and Technology, Wuhan, Hubei 430030, China

## Abstract

**Background:**

Ovarian cancer (OC) is the most fatal gynecologic cancer. The branched-chain *α*-keto acid dehydrogenase kinase (BCKDK) plays an important role in many serious human diseases, including cancers. Its function in promoting cell proliferation and migration has been reported in various cancers. However, the biological role of BCKDK and its molecular mechanisms underlying OC initiation and progression are unclear.

**Methods:**

First, the expression level of BCKDK in OC cell lines or tissues was determined using tissue microarray- (TMA-) based immunohistochemistry or western blotting. Then, growth curve analysis, anchorage-independent cell transformation assays, wound healing assays, cell migration assays, and tumor xenografts were used to test whether BCKDK could promote cell transformation or metastasis. Finally, the signaling pathways involved in this process were investigated by western blotting or immunoprecipitation.

**Results:**

We found that the expression of BCKDK was upregulated in OC tissues and the high expression of BCKDK was correlated with an advanced pathological grade in patients. The ectopic overexpression of BCKDK promoted the proliferation and migration of OC cells, and the knockdown of BCKDK with shRNAs inhibited the proliferation and migration of OC ex vivo and *in vivo*. Moreover, BCKDK promoted OC proliferation and migration by activating MEK.

**Conclusions:**

Our results demonstrate that BCKDK promotes OC proliferation and migration by activating the MEK/ERK signaling pathway. Targeting the BCKDK-MEK axis may provide a new therapeutic strategy for treating patients with OC.

## 1. Introduction

Among women over 40 years old, ovarian cancer (OC) is the deadliest and the second most common gynecological tumor after breast cancer [1, 2]. Because of the asymptomatic nature of OC, it is difficult to diagnose in the early stage, and most OC patients are initially diagnosed at an advanced stage.

At present, OC screening mainly relies on OC biomarker screening, such as serum cancer antigen 125 (CA125) or human epididymis protein. However, a randomized controlled trial involving more than 200,000 women showed that the mortality rate was not significantly reduced in the screening group compared with the nonscreening group [3]. Therefore, the fatality rate of OC has not decreased significantly in the past 30 years [1, 4]. Studies have shown that the use of PARP1 inhibitors (niraparib, olaparib, and rucaparib) significantly improves the progression-free survival of patients with OC [5–8]. The phenomenon of inherent or acquired resistance exists in OC patients, and PARP inhibitor therapy may not continue to be effective for all patients [8–10]. Therefore, new alternative or complementary-targeted drugs are still urgently needed.

Branched-chain *α*-keto acid dehydrogenase kinase (BCKDK), located in the mitochondrial matrix, belongs to the pyruvate dehydrogenase kinase (PDK) family [11], promotes the proliferation and metastasis of various tumors, and is considered to be a strong therapeutic target for preventing tumor development [12–17]. Dysfunction of BCKDK is closely related to various human diseases, especially maple syrup urine disease. McConnell et al. found that excessive activity of BCKDK resulted in maple syrup urine disease [18–20]. People with diabetes have increased susceptibility to OC, and they are always initially diagnosed in the late stages and have a poor prognosis [21]. Overexpression of BCKDK resulted in a branched-chain amino acid (BCAA) increase, and elevated plasma levels of BCAA were associated with a greater than 2-fold increased risk of future pancreatic cancer diagnosis [22]. Leucine promoted adipose tissue protein synthesis through the mTOR pathway [23], and adipocytes promoted OC metastasis and provided energy for rapid tumor growth [24]. BCKDK promoted colorectal cancer and hepatocellular carcinoma metastasis and proliferation via the ERK signaling pathway [25–27]. Furthermore, BCKDK was highly expressed in DOX-induced OC drug-resistant cell lines, and its expression level was twice as high as that of sensitive cell lines [28]. Inhibition of BCKDK increased the sensitivity of OC cells to paclitaxel [29]. Therefore, we hypothesized that BCKDK could promote OC proliferation and metastasis. In addition, we investigated which pathways regulated this process.

In this study, we showed that BCKDK had higher expression in OC patients than in adjacent tissues. High expression of BCKDK was correlated with advanced pathological grade in patients. Overexpression of BCKDK increased the colony formation and migration ability of SKOV3 and OVCAR3 cells ex vivo. Knockdown of BCKDK inhibited OC tumor progression *ex vivo* and *in vivo*. We identified BCKDK as an upstream kinase of MEK that upregulated MEK/ERK signaling by interacting with MEK. The above results suggested that BCKDK may promote OC proliferation and migration by enhancing the MEK/ERK signaling pathway.

## 2. Material and Methods

### 2.1. Plasmids, shRNA, Antibodies, and Other Reagents

The plasmids of pCMV-C-Flag (catalog: D2632) and pDONR223-BCKDK (catalog: 23794) were purchased from Beyotime Biotechnology (Shanghai, China) and Addgene (Cambridge, MA, USA), respectively. Then, the plasmids of pCMV-BCKDK-Flag and pLKO.1-shBCKDK were constructed by our laboratories. Two sequences were designed to knock down BCKDK, and the sequences were as follows: 1.5′-CCGGTCAGGACCCATGCACGGCTTTCTCGAGAAAGCCGTGCATGGGTCCTGATTTTTG-3′ and 2.5′-C CGGACGCTGACTTCGAGGCTTGGACTCGAGTCCAAGCCTCGAAGTCAGCGTTTTTTG-3′. A mock shRNA with a sequence lacking significant homology to the human genome database was used as the mock shRNA. The sequence was 5′-CCGGCCTAAGGTTAAGTCGCCCTCGCTCGAGCGAGGGCGACTTAACCTTAGGTTTTTG-3′. The sense and antisense oligonucleotides were synthesized, annealed, and cloned into the pLKO.1-TRC cloning vector at the *EcoRI* and *AgeI* sites as described by the manufacturer [30].

Anti-p-MEK1/2 (ser221) (catalog: 2338), anti-t-MEK (catalog: 8727), anti-phospho-p44/42MAPK (Erk1/2) (T202/Y204) (D13.14.4E) (catalog: 4370), and anti-t-ERK (catalog: 4695) antibodies were purchased from Cell Signaling Technology (Danvers, MA, USA). Anti-mouse BCKDK antibody (catalog: sc-374425) and anti-*β*-actin antibody (catalog: sc-130656) were purchased from Santa Cruz Technology, Inc. (Santa Cruz, CA, USA). The HRP-conjugated anti-rabbit (catalog: E030120) antibody and HRP-conjugated anti-mouse antibody (catalog: E030110) were purchased from EarthOx Life Sciences (San Francisco, CA, USA). Anti-Flag antibody (catalog: F1804, catalog: F7425) was purchased from Sigma-Aldrich (St. Louis, MO, USA). The DAB Detection Kit (Polymer) (catalog: PV-6000-D) was purchased from Zhongshan Golden Bridge Biotechnology Co., Ltd (Beijing, China). G418 (catalog: A1720) and puromycin were purchased from Sigma-Aldrich (St. Louis, MO, USA) and used for setting up stable cell lines. Simple-Fect (catalog: Profect-01) was purchased from Signaling Dawn Biotech (Wuhan, Hubei, China) for transfection.

### 2.2. Cell Culture

The human epithelial ovarian cancer cell lines (HO8910, HO8910-PM, SW626, SKOV3, and OVCAR3) and the human epithelial cell line (IOSE80) were purchased from American Type Culture Collection (ATCC; Manassas, VA, USA). IOSE80 cells were cultured in Dulbecco's Modified Eagle's Medium (Gibco, Gaithersburg, MD, USA) supplemented with 10% fetal bovine serum (FBS) (Gibco, Gaithersburg, MD, USA) and 2 mmol/L glutamine (Gibco, Gaithersburg, MD, USA). HO8910 and HO8910-PM cells were cultured in RPMI-1640 (Gibco) supplemented with 10% FBS and 2 mmol/L glutamine. SKOV3 cells were cultured in McCoy's 5A (Gibco) supplemented with 10% FBS and 2 mmol/L glutamine. OVCAR3 were cultured in RPMI-1640 (Gibco) supplemented with 20% FBS and 2 mmol/L glutamine. All cells were grown in 5% CO_2_ with saturated humidity at 37°C.

### 2.3. Western Blot

The cells (0.8 − 2 × 10^6^) were cultured in 10 cm diameter dishes to 70-80% confluence and starved without serum for 24 h. Then, the cells were treated with 40 ng/mL epidermal growth factor (EGF) (R&D catalog: 236-EG-200) for 15 min. EGF is a well-known tumor promotion agent used to study malignant cell transformation in animal and cell models of cancer [31]. After this, the cells were washed twice in PBS before being lysed in RIPA buffer (Coolaber, China). Furthermore, the samples were sonicated three times, with intervals of 15 seconds, and insoluble debris was removed by centrifugation at 13000 rpm for 15 min. Protein content was determined by BCA method (Coolaber, China). In addition, the protein within 30-120 *μ*g was separated by 10% SDS-PAGE and visualized by chemiluminescence (BIO-RAD, USA) in triplicate.

### 2.4. Growth Curve Analysis

The 2 × 10^5^ cells were plated in each dish and counted at different times in triplicate, using a hemacytometer to generate a growth curve.

### 2.5. Anchorage-Independent Cell Transformation Assay

Different cell lines (8 × 10^3^/well) were seeded into 6-well plates. The cells were then cultured in 1 mL of agar (Sigma-Aldrich Corp.) containing 10% FBS, 0.33% BME (Eagle basal medium, Sigma-Aldrich Corp.), 25 *μ*g/mL gentamicin, and 2 mM L-glutamine, with an additional 3 mL of 2 mM L-glutamine, 0.5% BME agar containing 10% FBS, and 25 *μ*g/mL gentamicin. Then, the cells were maintained in a 37°C, 5% CO_2_ incubator for 7-14 days, and the colonies were observed and assessed by microscopy in triplicate assays.

### 2.6. Bioinformatics

The Human Protein Atlas online database (https://www.proteinatlas.org/) was used to compare BCKDK mRNA expression in human normal tissues and human tumors. The mRNA expression data in normal tissues was indexed from Consensus dataset, HPA dataset, and GTEx database while in tumors was indexed from TCGA database. The gene name BCKDK could be used as search parameter in the searching toolbar, and all other parameters were set with default. BCKDK DNA alterations such as mutation, CNV gain, and CNV loss in different kind of tumors were analyzed in GDC online database (https://portal.gdc.cancer.gov/) by searching its gene name BCKDK in Quick Search toolbar, and other parameters were set with default.

### 2.7. RNA Sequencing

Three pairs of HO8910-PM-shMock and HO8910-PM-shBCKDK cells were used for transcriptomic analysis. Total RNA was extracted using TRIzol reagent (Thermo Fisher SCIENTIFIC, MA, USA). RNA sequencing was conducted by Jiangxi HyploX Medical Laboratory Co., Ltd. (Shangrao, China) with the Illumina PE150 platform. The genes with an absolute fold change ≥ 2 and an adjusted *p* value < 0.05 in expression were considered as the differentially expressed genes and were subsequently mapped onto KEGG analysis.

### 2.8. Tumor Xenografts

Female athymic Balb/c nude mice (4–6-week-old) were purchased from Chongqing Tengxin Beer Experimental Animal Sales CO, LTD (Chongqing, China). The mice were kept in specific pathogen-free conditions according to the National Guidelines for Animal Usage in Research (set by the Chinese government) at the Chongqing Population and Family Planning Science and Technology Research Institute. The mice were divided into three groups randomly. Each of the different cell lines (3 × 10^6^ in 200 *μ*L PBS) was injected subcutaneously into the right flank. And the tumor volume was measured every three days and were calculated with the formula V = 0.52 (length × width × height). Forty days after the cells injection, the mice were sacrificed with dislocation of cervical vertebra after injecting pentobarbital sodium (50 mg/kg, i.p.), 5 min. The tumor tissues were prepared with paraffin sections after fixation with formalin and then stained with hematoxylin and eosin (H&E).

### 2.9. Patient Samples and Immunohistochemistry (IHC)

The tissue microarrays (TMA) of OC (catalog: FOV 1006) were purchased from Xi'an Tabos Bio Co., Ltd. This study was approved by the ethical committee of Yichang Second People's Hospital. The samples were obtained with informed consent before surgery. The TMA was stained following the standard protocol for IHC staining. High pressure repair was conducted with Tris/EDTA buffer (pH 10.0) for 120°C, 5 min. The sections were incubated with primary antibody against BCKDK (1 : 50). The DAB Detection Kit (Polymer) was used as the secondary detection system. Positive staining of brownish yellow particles was located in the cytoplasm. According to the immunoscores of Remmele scoring method [32], the scores greater than 2 were used as positive group, and the others were used as negative group. And the slides were independently examined by two pathologists.

### 2.10. Wound Healing Assay

The wound healing assays were applied to determine the migration ability of cells. The 2 × 10^5^ cells were cultured in a six-well plate until 80–90% confluence and then carefully scratched with a 10 *μ*L pipette tip. After washing three times with 1 × PBS to remove detached cells, images in 10 different wound fields were captured at respective time points (0 h and 48 h) to evaluate the migration of cells in triplicate assays.

### 2.11. Transwell Assay

Chambers (catalog: 3422, 8 *μ*m pore, Corning, NY, USA) were used to investigate migration ability of cells. Initially, the 1 × 10^5^ cells suspended in 150 *μ*L serum-free medium were seeded onto the upper chamber of 24-well plates, and 700 *μ*L of medium with 10% FBS was added to the lower chamber. 48 h later, the medium was removed from the upper chamber. The nonmigrating cells on the upper side of the chamber were removed thoroughly with a clean cotton swab. Whereafter, the cells on the bottom side of the membrane were fixed with 4% paraformaldehyde for 30 min and then stained with 0.1% of crystal violet (Sangon Biotech) for 15 min. Finally, the stained cells were counted by microscopy. The results represent the average number of cells in three fields per membrane in triplicate inserts.

### 2.12. Immunoprecipitation

HO8910-PM were seeded in 10 cm dishes for 24 h. Then, the cells were harvested in IP buffer (150 mM NaCl, 50 mM Tris-HCl pH 7.4, 1% NP40, 1 mM DTT, and 1 mM EDTA). The amount of 2 mg proteins was subjected to immunoprecipitation following the manufacturer's instruction (https://www.scbt.com/protocols.html?protocol=immunoprecipitation). The mouse source antibody was used for IP, and the rabbit source antibody was used for western blotting.

### 2.13. Statistical Analysis

All quantitative data in the present study were performed at least in triplicate. Statistical analysis was examined using IBM SPSS Statistics 22.0. The results are expressed as the mean ± standard deviation. A two-tailed ANOVA or Student's *t*-test was used to evaluate the data. The associations between the level of BCKDK and the clinicopathological parameters of the GC patients were determined by using Pearson correlation coefficients. And *P* < 0.05 was considered significant (∗*p* < 0.05, ∗∗*p* < 0.01, and ∗∗∗*p* < 0.001).

## 3. Results

### 3.1. BCKDK Is Highly Expressed in OC and Is Associated with Pathological Grading of OC Patients

**Figure 1:**
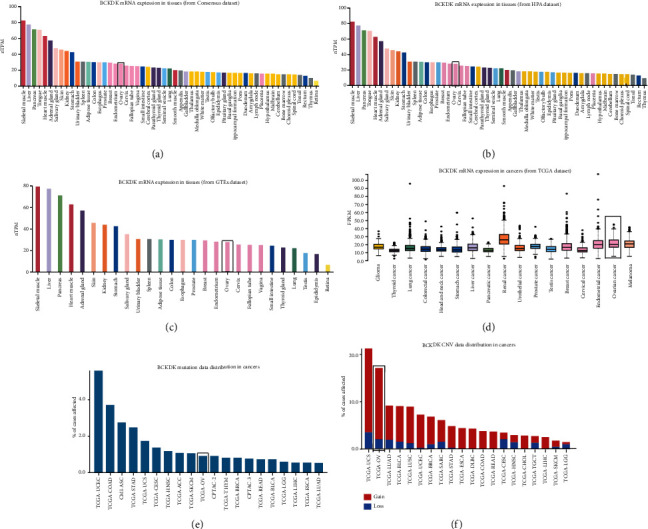
Distribution of BCKDK mRNA in normal tissues and cancers. (a–c) BCKDK mRNA expression levels in normal tissues indexed from Consensus dataset, HPA dataset, and GTEx database, respectively. (d) BCKDK mRNA expression levels in cancers indexed from TCGA dataset. (e–f) GDC online database was used to detect the DNA alterations of BCKDK in different human tumors, and data were indexed from TCGA database. Ovarian cancer had the second highest DNA amplification of BCKDK among these cancers tested. Red bar represents CNV gain, and blue bar represents CNV loss. Rectangle marks normal ovarian tissue data or ovarian cancer.

**Figure 2:**
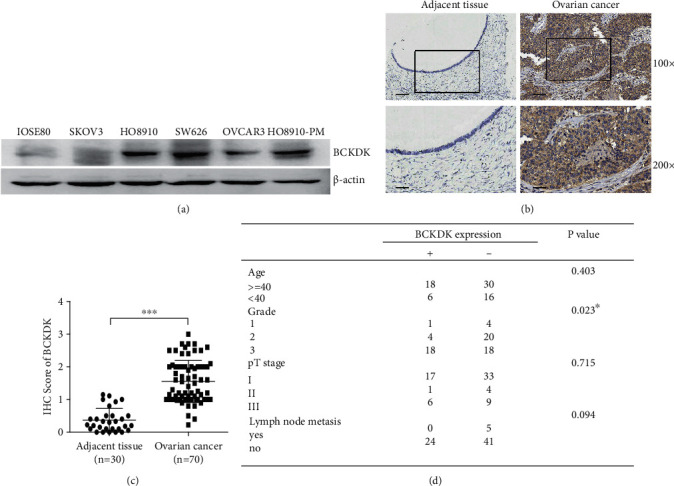
BCKDK overexpression is associated with advanced pathological grade in OC patients. (a) Expression of BCKDK in 6 different ovarian cell lines. (b) Immunohistochemical examination for the expression of BCKDK in 70 cases of human ovarian cancer tissues and 30 of adjacent tissues. Pictures from 1 representative case are shown in the *upper panel*, and the 2 scale bars from up to down in each group correspond to 100 and 40 *μ*m, respectively. (c) Statistics of the IHC examination results are shown. The IHC score of BCKDK is higher in OC tissues than adjacent tissues. (d) The correlation between BCKDK expression and clinicopathologic features and correlation data were determined by using Pearson's correlation coefficients. Error bars represent the mean ± SD values (∗∗∗*P* < 0.001).

Based on the Human Protein Atlas online database analysis, this study showed that normal ovarian tissues had weak or low expression of BCKDK among normal human tissues, while ovarian cancer had one of the highest mRNA expression levels of BCKDK among tumors (Figures 1(a)–1(d)). The GDC online dataset contains mutation data and DNA Copy Number Variation (CNV) of tumor samples from TCGA database (Figures 1(e) and 1(f)). BCKDK DNA is amplified in 15.21% tumors of ovarian cancer patients, which is the second highest proportion just lower than that of uterine carcinosarcomas of 17.86% (Figure 1(f)). In addition to this, BCKDK expression levels were detected in 1 normal epithelial ovarian cell line and 5 OC cell lines (Figure 2(a)). The results showed that the BCKDK level of IOSE80 cells was the lowest. BCKDK was poorly expressed in SKOV3 and OVCAR3 cells and highly expressed in HO8910, HO8910-PM, and SW626 cells. Then, the expression level of BCKDK was also determined in OC tissue and corresponding tumor adjacent tissue samples. The results demonstrated that the expression level of BCKDK was higher in OC tissues than in corresponding adjacent tissues (Figures 2(b) and 2(c)) and was associated with the pathological grading of patients (Figure 2(d)).

### 3.2. BCKDK Promotes OC Cell Proliferation

**Figure 3:**
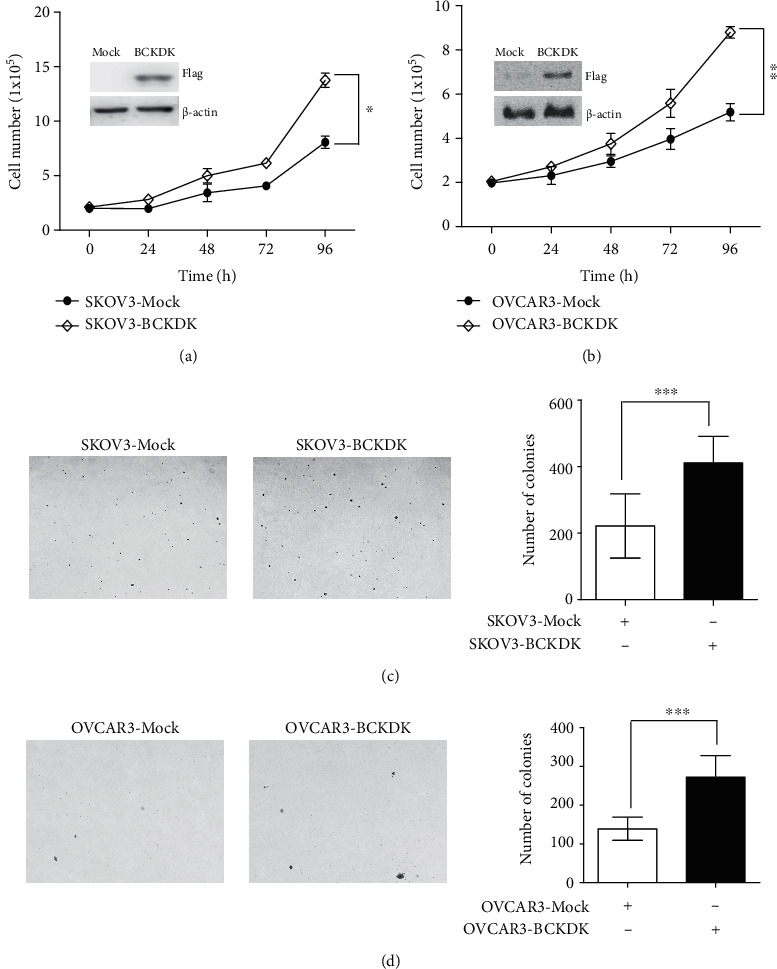
BCKDK promotes OC cell proliferation. (a) Growth curves of vector control cells (SKOV3-Mock) and BCKDK-overexpressing cells (SKOV3-BCKDK). Insert shows verification of the cell lines identified by western blot. Data are represented as mean ± standard deviation from the triplicate experiments. The asterisk indicates a significant increase in cell number in SKOV3-BCKDK cells compared with SKOV3-Mock cells (∗*P* < 0.05). (b) Growth curves of vector control cells (OVCAR3-Mock) and BCKDK-overexpressing cells (OVCAR3-BCKDK). Insert shows verification of the cell lines identified by western blot. Data are represented as mean ± standard deviation from the triplicate experiments. The asterisk indicates a significant increase in cell number in OVCAR3-BCKDK cells compared with OVCAR3-Mock cells (∗∗*P* < 0.01). (c) BCKDK can transform SKOV3 cells ex vivo as illustrated by growth of BCKDK transformed cells in soft agar. Photomicrograph of representative colony formation in soft agar of vector control cells (SKOV3-Mock) compared with BCKDK-overexpression cells (SKOV3-BCKDK) is shown (∗∗∗*P* < 0.001). (d) BCKDK can enhance the transformation of OVCAR3 cells *ex vivo* as illustrated by growth of BCKDK transformed cells in soft agar. Photomicrograph of representative colony formation in soft agar of vector control cells (OVCAR3-Mock) compared with BCKDK-overexpressing cells (OVCAR3-BCKDK) is shown (∗∗∗*P* < 0.001).

To test whether BCKDK can promote cell proliferation, BCKDK was overexpressed in SKOV3 and OVCAR3 cells, which poorly expressed BCKDK. SKOV3 and OVCAR3 stable cell lines were generated by transfecting the pCMV-c-Flag or pCMV-BCKDK-Flag plasmid into cells, and the growth curves of SKOV3-Mock or SKOV3-BCKDK cells were compared. The results demonstrated that SKOV3-Mock cells grew slower than SKOV3-BCKDK cells (Figure 3(a), inner section indicating BCKDK overexpression). Then, the anchorage-independent growth of SKOV3-Mock or SKOV3-BCKDK cells was also compared, and the results showed that the number of colonies in SKOV3-Mock cell cultures was less than that in SKOV3-BCKDK cell cultures (Figure 3(c) left panel). The corresponding statistical significance is indicated in the right panel of Figure 3(c). Similar results were observed in the cultures of OVCAR3-Mock or OVCAR3-BCKDK stable cells (Figures 3(b) and 3(d)). These results indicated that BCKDK promoted OC cell proliferation.

### 3.3. BCKDK Promotes OC Cell Migration

**Figure 4:**
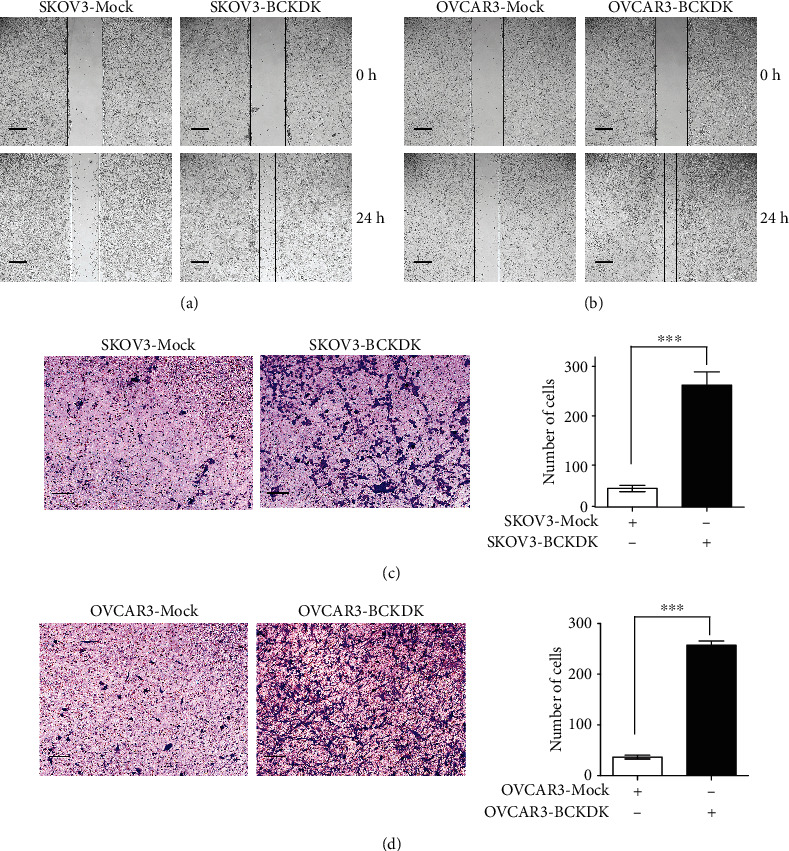
BCKDK promotes OC cell migration. (a) Scratch wound assay demonstrating that SKOV3-Mock migrate faster than SKOV3-BCKDK cells. The dotted lines show the area where the scratch wound was created. The scratch wound assay was performed in triplicate experiments. (b) Scratch wound assay demonstrating that OVCAR3-Mock migrate faster than OVCAR3-BCKDK cells. The dotted lines show the area where the scratch wound was created. The scratch wound assay was performed in triplicate experiments. (c) Transwell assay. SKOV3-BCKDK have greater migration capacity than SKOV3-Mock cells. Representative images from transwell assays of SKOV3-Mock cells and SKOV3-BCKDK cells are shown. (d) Transwell assay. OVCAR3-BCKDK have greater migration capacity than OVCAR3-Mock cells. Representative images from transwell migration assays of OVCAR3-Mock cells and OVCAR3-BCKDK cells are shown (∗∗∗*P* < 0.001).

Since BCKDK is closely associated with tumor migration in colorectal cancer [26], we investigated whether BCKDK also regulated OC migration. To test this hypothesis, wound healing assays and transwell assays were used to detect the effects of BCKDK on the migration of SKOV3 or OVCAR3 cells. The wound healing assay results are shown in Figures 4(a) and 4(b), which demonstrated that SKOV3/OVCAR3-Mock cells had weaker wound healing ability than SKOV3/OVCAR3-BCKDK cells. Furthermore, transwell assay results indicated that fewer SKOV3/OVCAR3-Mock cells migrated from the upper chamber than SKOV3/OVCAR3-BCKDK cells. Overexpression of BCKDK accelerated the migration of SKOV3 and OVCAR3 cells (Figures 4(c) and 4(d)). Therefore, the results above suggested that overexpression of BCKDK accelerated the migration capability of OC cells.

### 3.4. Knockdown of BCKDK Attenuates OC Tumor Properties

**Figure 5:**
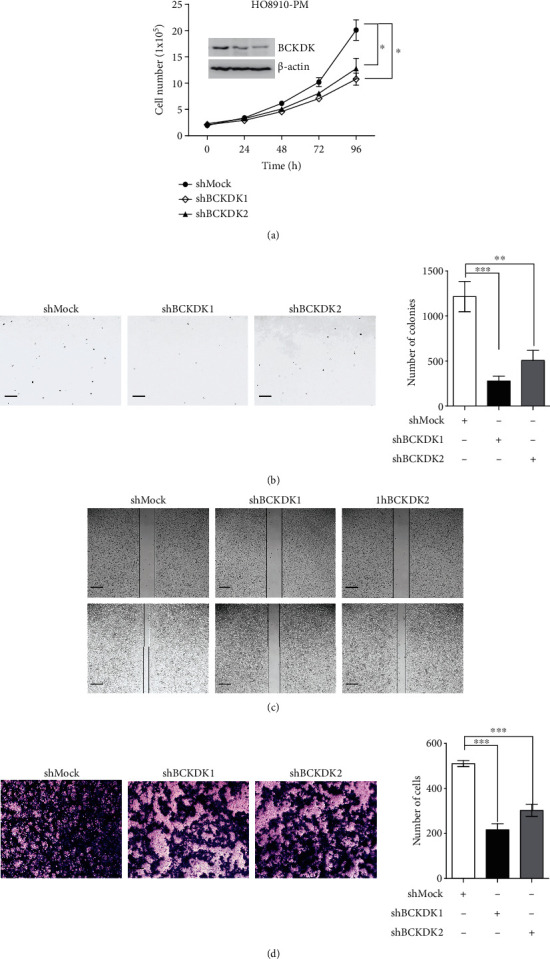
Knockdown of BCKDK attenuates OC tumor properties *ex vivo*. (a) Growth curves of HO8910-PM-shMock, HO8910-PM-shBCKDK1, and HO8910-PM-shBCKDK2 cells. Insert shows verification of the knockdown cell lines identification by western blot. Data are represented as mean ± standard deviation from triplicate experiments. The asterisks indicate a significant increase compared with shMock cells (∗*P* < 0.05). (b) Knockdown of BCKDK reduces tumorigenic properties of HO8910-PM cells *ex vivo*. Representative photomicrograph of colony formation in soft agar of vector control cells (shMock) compared with BCKDK-knockdown cells (shBCKDK1 or shBCKDK2) is shown. Data are represented as mean ± standard deviation from triplicate experiments (∗∗*P* < 0.01 and ∗∗∗*P* < 0.001). (c) Scratch wound assay demonstrating that HO8910-PM-shMock migrate faster than HO8910-PM-shBCKDK1, 2 cells. The dotted lines show the area where the scratch wound was created. The scratch wound assay was performed in triplicate experiments. (d) Transwell assay. HO8910-PM-shMock have greater migration capacity than HO8910-PM-shBCKDK1, 2 cells. Representative images from transwell assays of HO8910-PM-shMock cells and HO8910-PM-shBCKDK1, 2 cells, are shown.

**Figure 6:**
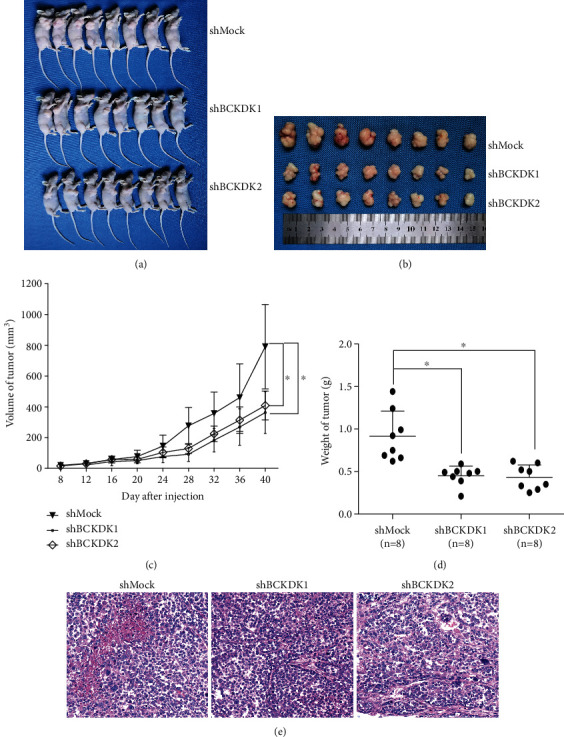
Knockdown of BCKDK reduces tumorigenic properties of HO8910-PM cells *in vivo*. (a) Mice and (b) tumors dissected from each group are shown. (c) Final average tumor growth curve and (d) tumor weight of mice injected with HO8910-PM-shMock or HO8910-PM-shBCKDK cells are shown. Data are shown as means ± standard deviation of measurements. The asterisk indicates a significant decrease in tumor size of HO8910-PM-shBCKDK-injected mice compared with HO8910-PM-shMock-injected mice (∗*P* < 0.05). (e) Immunohistochemistry analysis was performed in the tumor tissues of HO8910-PM-shMock-injected mice or HO8910-PM-shBCKDK-injected mice.

To further verify the above results, BCKDK was knocked down in HO8910-PM OC cells to generate stable shMock cell lines and stable shBCKDK cell lines (HO8910-PM-shMock and HO8910-PM-shBCKDK). The result in the inner section of the *left panel* in Figure 5(a) shows that BCKDK was knocked down by shRNA sequences in lines 1 and 2. Growth curves of HO8910-PM-shMock or shBCKDK cell lines were tested. The results indicated that HO8910-PM-shMock cells grew dramatically faster than HO8910-PM-shBCKDK cells (Figure 5(a)). Then, the anchorage-independent growth of the HO8910-PM-shMock or shBCKDK cell lines was analyzed, and the results demonstrated that the number of colonies in HO8910-PM-shMock cell cultures was much greater than that in HO8910-PM-shBCKDK cell cultures (Figure 5(b)). Wound healing assays and transwell assays of the HO8910-PM-shMock or HO8910-PM-shBCKDK cell lines were also performed, and the results suggested that knockdown of BCKDK attenuated the migration of HO8910-PM cells (Figures 5(c) and 5(d)). Therefore, these above results indicated that knockdown of BCKDK in OC cells inhibited tumorigenesis and migration ex vivo. Furthermore, tumor xenograft assays were also performed in female athymic Balb/c nude mice. We injected HO8910-PM-shMock or HO8910-PM-shBCKDK cells (3 × 10^6^) subcutaneously into the right flank, and tumor size was assessed over 40 days. Tumors in HO8910-PM-shBCKDK-inoculated mice grew to a smaller size than those in HO8910-PM-shMock-inoculated mice (Figures 6(a) and 6(b)). In addition, the tumor growth curve is shown in Figure 6(c). The final weight of tumor is shown in Figure 6(d). The tumor tissues dissected from these xenografts in the study were stained with hematoxylin and eosin (H&E) to verify that these tissues belong to tumor tissues (Figure 6(e)). These data indicated that inhibiting BCKDK expression in OC cells significantly weakened their tumorigenic properties *ex vivo* and *in vivo* and further verified that BCKDK promotes OC cell proliferation and migration.

### 3.5. BCKDK Promotes Tumor Properties by Upregulating the MEK-ERK Signaling Pathway

**Figure 7:**
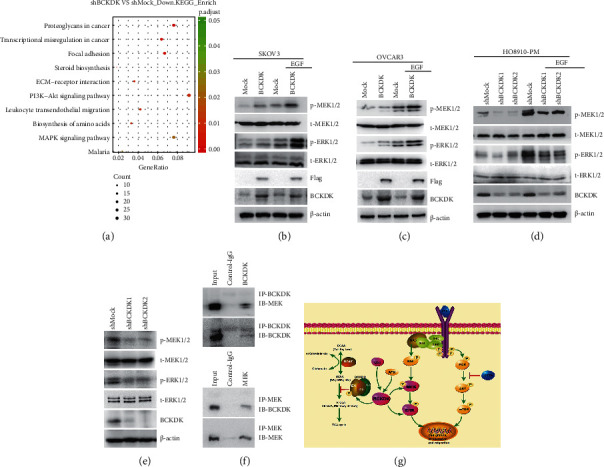
BCKDK promotes tumorigenesis through upregulating MEK-ERK signaling pathway. (a) Bubble chart of KEGG pathway analysis figured out the MAPK pathway associated with BCKDK expression. (b and c) The level of phosphorylation of MEKs and ERKs was increased in SKOV3/OVCAR3-BCKDK cells after EGF treatment for 15 min. (d) The level of phosphorylation of MEKs and ERKs was decreased in HO8910-PM-shBCKDK cells after EGF treatment for 15 min. (e) The level of phosphorylation of MEKs and ERKs was decreased in the tumor tissues from HO8910-PM-shBCKDK-injected mice compared to HO8910-PM-shMock-injected mice. (f) BCKDK binds with MEK in HO8910-PM cells. Endogenous BCKDK or MEK was immunoprecipitated from HO8910-PM cells and then probed with anti-MEK or anti-BCKDK antibody. All western blot data are representatives of results from triplicate experiments. (g) The signaling pathway of BCKDK in cancers [26–28].

We confirmed that BCKDK promoted cell proliferation and migration *ex vivo* and *in vivo*. Next, we investigated which signaling pathway was involved in this process. BCKDK promotes carcinogenesis in colorectal cancer and hepatocellular carcinoma, and the MAPK signaling pathway functions in this process [25, 27]. And transcriptomic analysis was utilized to show that MAPK signaling pathway was also one of the most enriched pathways associated with BCKDK expression (Figure 7(a)). Therefore, p-MEK1/2 (Ser221) and p-ERK1/2 (T202/Y204) levels were detected in OVCAR3 or SKOV3 stable cell lines, and the results demonstrated that the levels of p-MEK1/2 (Ser221) and p-ERK1/2 (T202/Y204) were elevated when BCKDK was overexpressed (Figures 7(b) and 7(c)). These results suggested that BCKDK promoted OC by upregulating MEK-ERK activity. Then, the levels of BCKDK, p-MEK1/2 (Ser221), and p-ERK1/2 (T202/Y204) were also determined in the HO8910-PM-shBCKDK cell lines. Both p-MEK1/2 (Ser221) and p-ERK1/2 (T202/Y204) were dramatically decreased when BCKDK was knocked down in HO8910-PM cell lines (Figure 7(d)). Furthermore, the expression levels of BCKDK, p-MEK1/2 (Ser221), and p-ERK1/2 (T202/Y204) were tested in dissected tumor tissues. The results confirmed that the levels of BCKDK, p-MEK1/2 (Ser221), and p-ERK1/2 (T202/Y204) were lower in the tumor tissue of HO8910-PM-shBCKDK mice than in the tumor tissue of HO8910-PM-shMock mice (Figure 7(e)). These data confirmed that BCKDK promoted tumorigenic properties by upregulating the MEK-ERK signaling pathway.

### 3.6. BCKDK Interacts with MEK

We previously confirmed that BCKDK interacted with MEK in colorectal cancer cells [25]. Thus, we investigated whether BCKDK also interacted with MEK in OC cells. BCKDK was immunoprecipitated from HO8910-PM cells and detected with a MEK antibody by western blotting. The results demonstrated that BCKDK co-immunoprecipitated with MEK in HO8910-PM cells (Figure 7(f)).

Taken together, our study indicates that BCKDK promotes OC proliferation and migration by activating the MEK/ERK signaling pathway.

## 4. Discussion

OC is the deadliest gynecological tumor. The five-year survival rate of OC patients is as low as 15-45% [33], while the survival rate of OC patients in FIGO's stage I is as high as 90% or above. To improve the diagnosis of OC, a variety of serum markers have been developed and used in the diagnosis of OC patients. In the 1980s, CA125 was reported as a tumor marker for OC [34]. However, the sensitivity and specificity of these markers are not as high as expected [2, 35]. Therefore, there is still an urgent need to develop specific serum markers that can be screened without internal inspections. As there are no external manifestations before the advanced stage of OC patients, the fatality rate has not decreased significantly in the past 30 years [4, 36]. The new alternative or complementary targeted drugs also need to be researched and developed.

Previous studies demonstrated that the amino acid profile could be an effective diagnostic tool in various cancer patients [37–40], and some amino acids are associated with OC [41–44], for example, leu [45]. The catabolism of BCAA is closely related to the development and progression of various tumors. Inhibition of BCAA catabolism can promote tumor growth and development [46, 47]. Inhibiting the expression of BCAA catabolic enzymes can lead to the accumulation of BCAA in tumors, while the liver regeneration tissues were not accumulated [46]. Current research focuses on BCAT1 or BCAT2, which works in the first step of BCAA catabolism, while there are relatively few studies on BCKDK, which is a key negative regulatory enzyme in BCAA catabolism [48–51]. Despite this, studies have shown that the overexpression of BCKDK promotes the growth and metastasis of various tumors [25–27]. In this study, we determined that BCKDK promoted the proliferation and metastasis of OC, and BCKDK was expressed at higher levels in OC tissues than in adjacent normal tissues (Figure 2(b)) and is correlated with advanced pathological grade for patients (Figure 2(d)). This suggests that BCKDK could be another potential biomarker for the treatment of OC. Inhibitors targeting BCKDK will be examined in future research.

Current research of BCAA catabolism worked in tumors focused on BCAT. As the BCAT reaction is reversible and near equilibrium, its direction should respond to changes in concentrations of BCAA and BCKAs and availability of the donors and acceptors of nitrogen; to some extent, the conclusion was opposite in different researches. For example, some studies confirmed that the high expression of BCAT promoted the transfer of the BCAA amino group to *α*-ketoglutarate (*α*-KG) to form glutamate and the corresponding branched-chain keto acids (BCKAs). The BCAA catabolism was increasing, and then, the BCA-CoA entering into tricarboxylic acid cycle provided energy for tumor cells proliferation and growth [51, 52]. The other studies verified that the catabolism of BCAA in tumor cells was decreasing, and the high expression of BCAT promoted the conversion of BCKAs to BCAA and *α*-KG, providing essential nutrients and energy for cancer growth [46–48]. Our research supports the second opinion. The overexpression of BCKDK inhibits the conversion of BCKAs to BCA-CoA, which leads to the accumulation of BCKAs. Furthermore, the accumulation of BCKAs inhibits BCAA catabolism. Therefore, BCKAs are converted into BCAAs again through amination with the BCAT enzyme. In addition, there are studies showing that BCKDK and PPM1K make up a ChREBP-regulated node that integrates BCAA and lipid metabolism and promotes BCAAs as a material for fat synthesis for fat cells, which provide energy for tumor growth [53]. It has been proven that leucine was increased in OC [45]. Other studies also found that the overexpression of BCAT promoted OC proliferation [49–51]. Therefore, our research gave a further understanding of BCAA catabolism that worked in the OC and how BCKDK coordinated with BCAT to balance the BCAA metabolism. Whether they could directly regulate each other was still unclear.

Furthermore, to confirm the function of BCKDK in OC, BCKDK was overexpressed in SKOV3 and OVCAR3 cells which poorly expressed BCKDK. BCKDK gain significantly promoted the proliferation and migration ex vivo, whereas knocked down expression of BCKDK in HO8910-PM OC cells reduced the proliferation and migration *ex vivo* and inhibited the tumor growth *in vivo*. Hence, these data supported the tumor-promoting function of BCKDK in OC. These results were also consistent with the previous findings demonstrating that BCKDK is a key regulator of cell proliferation and metastasis in colorectal cancer and hepatocellular carcinoma [25–27]. Moreover, BCKDK promotes OC proliferation and migration by activating the MEK/ERK signaling pathway. In agreement with our previous study, which demonstrated that BCKDK promoted colorectal cancer proliferation by targeting the MEK1 [25]. Another study also verified that BCKDK promoted hepatocellular cancer proliferation by MEK/ERK signaling pathway [27]. To our knowledge, this study was the first to report the ectopic expression of BCKDK in OC and uncovered the mechanism that BCKDK regulates OC proliferation and migration by MEK/ERK signaling pathway.

Other studies also showed that BCKDK was related to lipid metabolism, which was upregulated by APN [27]. In addition, BCKDK promoted tumor growth and metastasis by interacting with SRC or mTOR in colon cancer or hepatic carcinoma (Figure 7(g)) [26, 27]. Therefore, in addition to the MEK-ERK pathway, whether the APN, SRC, or mTOR signaling pathways are also involved in this process still needs further examination. In addition, the drug resistance of OC is a thorny issue; the mitochondria are closely related to apoptosis and autophagy-induced drug resistance [54–56]. BCKDK is located in the mitochondria and is related to drug resistance of OC [28, 29]. Which pathways regulated this process? Many questions need to be addressed in the future. Because the follow-up data were missing, overall survival rates and progression-free survival rates were not analyzed in this study, and future studies need to collect more clinical information.

## 5. Conclusions

These findings indicated that the expression of BCKDK was upregulated in OC tissues and that high expression of BCKDK was correlated with advanced pathological grade in patients. BCKDK promoted OC proliferation and migration by activating MEK. Targeting the BCKDK-MEK axis may provide a new therapeutic strategy for treating patients with OC.

## Data Availability

The datasets generated and analyzed in the current study are available from the corresponding author upon reasonable request.

## Abbreviations

OC: Ovarian cancer
BCKDK: Branched-chain *α*-keto acid dehydrogenase kinase
CA125: Cancer antigen 125
PDKs: Pyruvate dehydrogenase kinases
BCAA: Branched-chain amino acid
ERK: Extracellular signal-regulated kinase
EGF: Epidermal growth factor
TMA: Tissue microarray
BME: Eagle basal medium
H&E: Hematoxylin and eosin
*α*-KG: *α*-Ketoglutarate
BCKAs: Branched-chain keto acids.
